# Creation of a Realistic Model for Removal of a Metallic Corneal Foreign Body for Less than $75

**DOI:** 10.5811/westjem.2016.10.32234

**Published:** 2016-12-15

**Authors:** Julie S. Sayegh, Sari Lahham, Logan Woodhouse, Jenny Seong, C. Eric McCoy

**Affiliations:** * University of California, Irvine, School of Medicine, Irvine, California; † University of California, Irvine, UC Irvine Medical Center, Orange, California

## BACKGROUND

Metallic corneal foreign bodies (MCFB) are one of the most common causes of ocular injury presenting to the emergency department.^1^–^4^ Patients are at risk of developing tissue necrosis, infection, and even vision loss if the foreign body is not removed in a timely manner.^1^–^3^,^5^,^6^ Traditionally, these foreign bodies are removed under slit-lamp examination using a sterile, large-gauge needle, followed by an electric burr to remove the subsequent rust ring.^1^,^3^,^5^–^7^ Forceful attempts to remove the MCFB can lead to perforation of the anterior chamber, corneal scarring, and worsening of vision.^1^,^6^,^7^ Healthcare providers must use proper technique in removing ocular foreign bodies to prevent these underlying complications.^1^,^6^,^7^ Several studies have shown that simulation improves procedural confidence and skill in MCFB removal.^4^,^6^,^7^ Models made from bovine eyes, agar plates, gelatin, and corneas created from glass and paraffin wax have previously been created; however, the use of corneas created from ballistics gel for MCFB removal has not been studied.^4^,^6^,^7^ We propose a realistic, sustainable, cost-effective MCFB task-trainer to introduce the fundamental skills required for MCFB removal.

This task-trainer also helps with the kinesthetics required for measurement of intraocular pressure (IP) with a Tono-Pen. In a brief PubMed search using the words Tono-Pen task-trainer, IP task-trainer, ocular glaucoma task-trainer, and IP simulator, only one study using a rubber glove filled with water was found to simulate this task.^8^

## OBJECTIVE

The objective of this article is to provide educators with an easy to follow, step-by-step recipe to create a realistic, sustainable, cost-effective MCFB and Tono-Pen task-trainer to train healthcare providers who will be responsible for patients requiring these procedures.

Creation of this task-trainer requires a total time of approximately 90 minutes, and costs less than $75 to create. This task-trainer is comprised of three major components: the head, eyeball and cornea.

## CURRICULAR DESIGN

### Items Needed

#### Head

12-inch Styrofoam mannequin head: $7–13 (amazon.com)
Liquid latex: 4 oz. bottle $6–10 (ebay.com)
11-blade scalpel/small, sharp knife: $1 (Dollar Tree/99 Cents Only Store)

#### Molds for Eyeball and Cornea

100% clear silicone tube: $3–6 (Home Depot)
1-pt paint thinner: $3–4 (Home Depot)
Baby oil: $2–4 (Walmart)
1 box of cornstarch: $1 (This item and items below: Dollar Tree/99 Cents Only Store)
Small ¾ inch plastic balls: $1
Bag of small, round 0.5-inch marbles: $1
Jar of petroleum jelly: $1
Disposable plastic cups: $1
Disposable plastic spoons: $1
Disposable latex gloves: $1

#### Cornea

1-lb block of ballistics gel: $14 (http://store.clearballistics.com)
0.5-inch circle hole punch: $5 (amazon.com, brand: EK tools)
Microwave-safe glass cup: $1 (This item and item below: Dollar Tree/99 Cents Only Store)
Instant glue: $1

#### Metallic Foreign body

Paper clips: $1/packet (This item and items below: Dollar Tree/99 Cents Only Store)
Tweezers: $1
Wire cutters: $1

### Assembly instructions

Begin creation of the task-trainer by preparing the head first so that ample time can be allowed for it to dry.

#### The Head

1. Take the 12-inch Styrofoam mannequin head, and using an 11-blade scalpel or sharp knife, cut out the eyes to create approximately one-inch deep eye sockets.
2. Paint the Styrofoam head with a thin coating of flesh-colored liquid latex, and set aside to dry.

After preparing the head, begin making the molds for the eyeball and cornea. Make sure to wear gloves prior to handling the silicone mixture. Also, ensure the work is done in a well-ventilated space as both silicone and paint thinner release a noxious odor. (See material data safety sheets [MSDS] for safety and handling information.) 9,10

### The Molds

#### Eyeball Mold

**Image 1:**
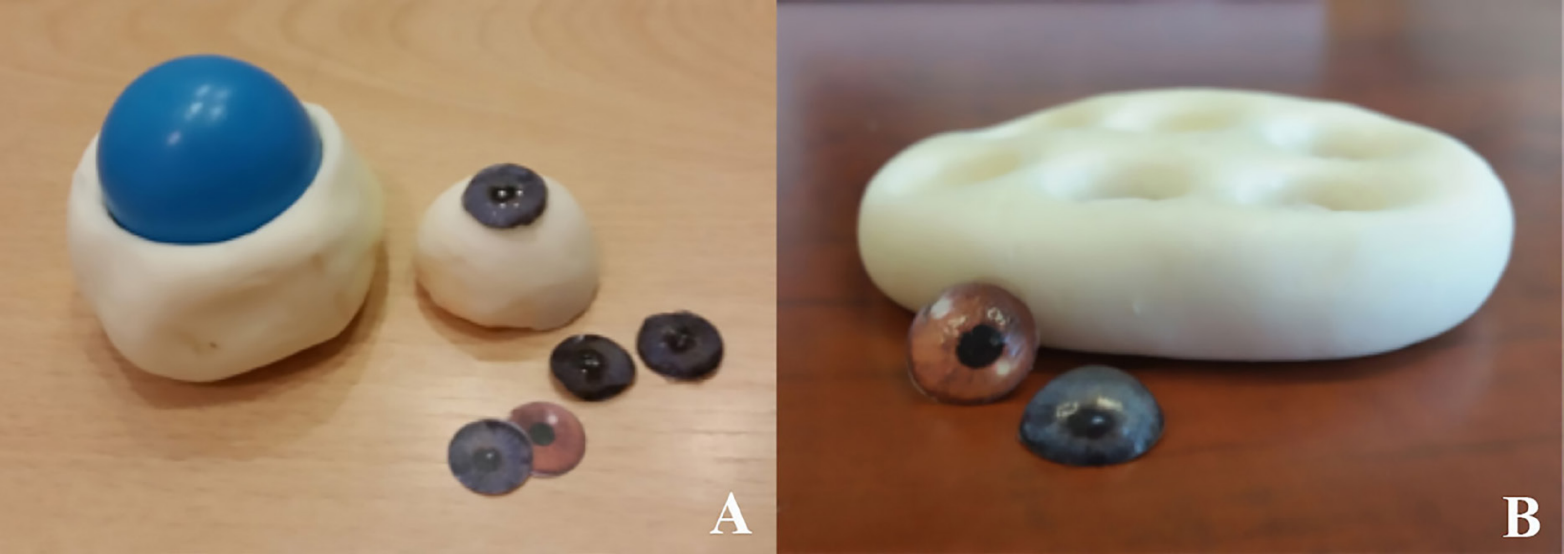
Mold for creating the eyeball, and finished eyebal with cornea, **1A**; and mold for creating the cornea, and completed cornea with iris, **1B.**

1. In a disposable plastic cup, squeeze ¼ cup of 100% clear silicone.
2. Add ¼ cup of cornstarch.
3. Add 5–6 drops of baby oil to the silicone/cornstarch mixture. Combine thoroughly using a disposable plastic spoon. The final mixture should have the consistency of dough.
4. Using gloved hands, remove the mixture from the plastic cup and form it into a ball.
5. Press a ¾-inch plastic ball halfway into the mold, making sure not to press the ball through the mold (Image 1A).
6. Allow the mold to cure for 20 minutes until firm.
7. After curing, remove the plastic ball from the mold.
8. Using a cleanly gloved finger, coat the impression of the ¾-inch ball with a thin layer of petroleum jelly. (This functions as a releasing agent for the material that will be placed inside to create the eyeball.)

#### Cornea Mold

1. Squeeze ¼ cup of 100% clear silicone into a disposable plastic cup.
2. Add ¼ cup of cornstarch.
3. Add 5–6 drops of baby oil to the silicone/cornstarch mixture, and combine thoroughly using a disposable plastic spoon. The final mixture should have the consistency of dough.
4. Using gloved hands, remove the mixture from the plastic cup and press it into a ¼-inch thick rectangle that is approximately 2 inches wide × 3 inches long. (Size may vary slightly depending on amount silicone/cornstarch mixture.)
5. Press 6 marbles, evenly spaced, halfway into the mold, ensuring not to press them through the mold.
6. Allow the mold to cure for 20 minutes until firm.
7. After curing, remove the marbles from the mold (Image 1B).
8. Using a cleanly gloved finger, coat the impressions from the marbles with a thin layer of petroleum jelly to act as a releasing agent for the cornea material.

### The Eyeball

**Image 2:**
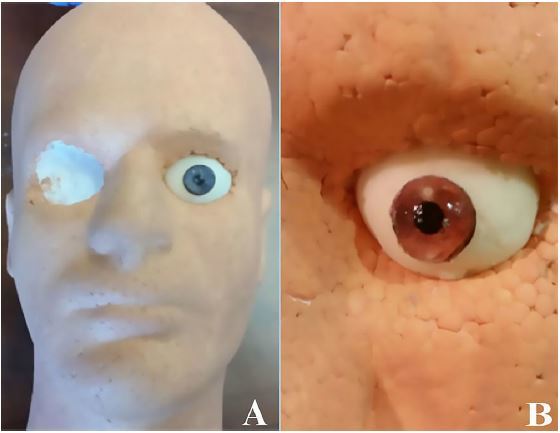
Completed task-trainer once assembled, **A**; and close-up image of the eye with embedded metallic corneal foreign body, **B**.

1. Squeeze ¼ cup of 100% clear silicone into a disposable plastic cup.
2. Add ¼ cup of cornstarch.
3. Rather than adding baby oil, add 2 tablespoons of paint thinner to the silicone/cornstarch mixture and mix thoroughly using a disposable plastic spoon. The paint thinner will soften the silicone, creating a more pliable and life-like feel to the eye. (Caution: this step releases a noxious smell; see MSDS for additional safety information.)^10^ The final mixture should have the consistency of pudding.
4. Fill the petroleum-lined eyeball mold with the eyeball mixture using the plastic spoon. Make sure to have gloves on during this step if not already wearing them.
5. Allow the eyeball mixture to cure for 15–20 minutes. (Once cured, the eyeball should feel firm. If not, allow to cure for an additional 5–10 minutes.)
6. After the curing process, apply lateral pressure to the eyeball to remove it from the mold.
7. Insert the eyeball into the hollowed-out eye socket on the Styrofoam mannequin head (Image 2A).
8. Repeat steps 1–7 to create the second eyeball.

### The Cornea

1. To create the iris for the cornea, obtain desired image of an iris from the Internet.
2. Resize the iris image to 0.5-inch using a printable document program. Paste several images of the iris on the same document and print it, preferably in color for a more life-like eye.
3. Using the 0.5-inch circular whole punch, cut out the irises and place aside (Image 1A).
4. Using the sharp knife or scalpel, cut a 1-inch cube of the clear ballistics gel and place into a microwave-safe glass cup.
5. Melt the ballistics gel in the microwave, using 2–4 minutes intervals until fully melted. (Do not stir or mix the ballistics gel until fully melted, as this will introduce bubbles into the gel and result in an unclear cornea).
6. Add 1 tablespoon of paint thinner to the melted ballistics gel and, using a plastic spoon, stir the solution slowly until thoroughly mixed, taking caution not to introduce bubbles into the mixture. (Caution: this step releases volatile gas so perform in a well-ventilated area).^10^
7. Slowly pour the mixture into the petroleum-lined cornea molds to prevent formation of bubbles.
8. Place a cut-out iris (from steps 1–3) over the top of each cornea, ensuring that the colored image side is downward. The paper will adhere to the ballistics gel, creating a cornea with its respective iris.
9. Allow corneas to harden for about five minutes.
10. Using a finger, apply gentle lateral pressure to each cornea to remove them from the mold (Image 1B).
11. Using instant glue, secure the cornea to the center of the eyeball that was placed inside the mannequin head (Image 2B). Take caution not to press the cornea too firmly as this will cause a deformity.

### The Metallic Foreign Body

Using wire cutters, cut a paperclip at a diagonal angle into 2-mm pieces to create a sharp MCFB.

1. Use the tweezers to embed the small metallic foreign body into the cornea at the desired location (Image 2B).
2. After being assembled, an18-guage needle attached to a syringe can be used to teach and practice the technique required to remove a MCFB, and to measure IP using a Tono-Pen.

A realistic click can be felt as the needle scrapes the MCFB. Due to the properties of the ballistics gel, the residual area of the MCFB will re-seal and can be reused multiple times as long as the needle tip does not destroy the gel. Once the task-trainer is created, only the corneas will need to be remade as needed, which should take only 15–20 minutes. The shelf life of unused corneas can be extended by several months when stored covered in the refrigerator, as the ballistics gel will dry out with time.

When using the Tono-Pen to measure IP, the pliability of the ballistics gel provides a realistic feel to the cornea, and leads to an accurate pressure reading of less than 20mmHg.

For increased realism, the mannequin head can be secured to a slit lamp using an IV tourniquet or strap. Additional props for realism, such as eyelashes, eyebrows or a wig, can also be applied.

## IMPACT/EFFECTIVENESS

We conducted an informal pilot study with the help of 14 learners – six medical students (MS) and eight emergency medicine residents – in order to assess the usefulness of this trainer. The learners were given surveys to determine their level of comfort performing MCFB removal and IP measurement with a Tono-Pen prior to, and after, using the task-trainer.

**Figure 1:**
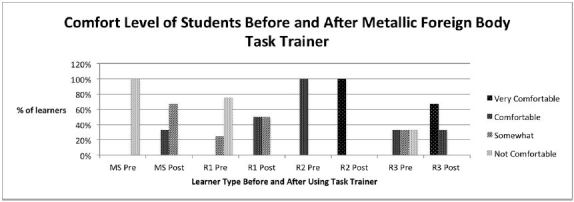
Level of comfort for medical students and residents year 1–3 before (pre) and after (post) the use of the metallic corneal foreign body task-trainer. *MS,* medical students, n=6; *R1*, first year residents, n=4; *R2*, second year residents, n=1; *R3*, third year residents, n=3.

**Figure 2:**
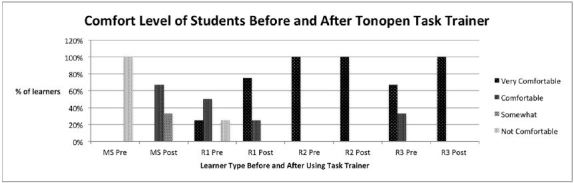
Level of comfort for medical students and residents year 1–3 before (pre) and after (post) the use of the Tono-Pen task-trainer. *MS*, medical students, n=6; *R1*, first year residents, n=4; *R2*, second year residents, n=1; *R3*, third year residents, n=3.

All of the MS group (100%) did not feel comfortable performing MCFB removal or using the Tono-Pen prior to using the task-trainer. After use, all (100%) felt at least somewhat comfortable or comfortable removing a MCFB and using the Tono-Pen (Figures 1 & 2).

Only 25% of the first-year residents (R1s) felt some degree of comfort removing a MCFB; however, after using the task-trainer, all felt some degree of comfort performing this procedure (Figure 1). For Tono-Pen use, 25% of R1s initially felt very comfortable using the Tono-Pen, with an increase to 75% after using the task-trainer (Figure 2).

All second-year residents (R2s) felt somewhat comfortable performing MCFB removal, and after using the task-trainer, all felt very comfortable performing this procedure (Figure 1). There was no change noted after using the Tono-Pen task-trainer, as all R2s initially felt very comfortable handling the Tono-Pen (Figure 2).

None of the third-year residents (R3s) felt very comfortable performing MCFB removal prior to using the task-trainer. However 67% felt very comfortable doing this procedure after task-trainer use (Figure 1). Seventy-five percent of R3s felt very comfortable using the Tono-Pen prior to using the task-trainer, which increased to 100% after use (Figure 2).

The learners were also asked to examine the task-trainer and comment on its realism. All learners felt that the task-trainer was realistic. Lastly, the learners were asked whether they felt this type of task-trainer would be valuable during their training, and all agreed that it would be valuable for both MCFB removal and measurement of IP with a Tono-Pen (Table).

As current trends in simulation become more focused on patient safety, task-trainers can provide an invaluable learning experience for residents, medical students and physicians.^6^,^11^,^12^ This task-trainer serves as a realistic, cost-effective, hands-on training tool that can improve the skills required to care for patients presenting with MCFBs, and can also teach the manual skills necessary for measuring intraocular pressures with a Tono-Pen. Creation of the task-trainer required a total time of 90 minutes. The cost of materials to build the model was less than $75.

## Figures and Tables

**Table:** Pilot study survey results: Survey results obtained for task-trainer after completion of metallic corneal foreign body removal and intraocular pressure measurements using a Tono-Pen; n=number of medical students or residents who participated in the survey.

	Metallic corneal foreign body		Tonopen	
		
	Medical students n=6	Residents n=8	Medical students n=6	Residents n=8
Have you performed this procedure before?	0/6 (0%)	2/8 (25%)	0/6 (0%)	7/8 (87.5%)
Do you think the task-trainer felt realistic?	6/6 (100%)	8/8 (100%)	6/6 (100%)	8/8 (100%)
Do you think this task-trainer is an effective tool for training?	6/6 (100%)	8/8 (100%)	6/6 (100%)	8/8 (100%)
